# A Review of Brain Activity and EEG-Based Brain–Computer Interfaces for Rehabilitation Application

**DOI:** 10.3390/bioengineering9120768

**Published:** 2022-12-05

**Authors:** Mostafa Orban, Mahmoud Elsamanty, Kai Guo, Senhao Zhang, Hongbo Yang

**Affiliations:** 1 School of Biomedical Engineering (Suzhou), Division of Life Sciences and Medicine, University of Science and Technology of China, Hefei 230026, China; 2 Suzhou Institute of Biomedical Engineering and Technology, Chinese Academy of Sciences, Suzhou 215163, China; 3 Mechanical Engineering Department, Faculty of Engineering at Shoubra, Banha University, Cairo 13518, Egypt; 4 Mechatronics and Robotics Department, School of Innovative Engineering Design, Egypt-Japan University of Science and Technology (E-JUST), Alexandria 21934, Egypt

**Keywords:** brain-computer interfaces (BCI), electroencephalogram (EEG), exogenous EEG signals, motor imaginary, SSVEP, P300

## Abstract

Patients with severe CNS injuries struggle primarily with their sensorimotor function and communication with the outside world. There is an urgent need for advanced neural rehabilitation and intelligent interaction technology to provide help for patients with nerve injuries. Recent studies have established the brain-computer interface (BCI) in order to provide patients with appropriate interaction methods or more intelligent rehabilitation training. This paper reviews the most recent research on brain-computer-interface-based non-invasive rehabilitation systems. Various endogenous and exogenous methods, advantages, limitations, and challenges are discussed and proposed. In addition, the paper discusses the communication between the various brain-computer interface modes used between severely paralyzed and locked patients and the surrounding environment, particularly the brain-computer interaction system utilizing exogenous (induced) EEG signals (such as P300 and SSVEP). This discussion reveals with an examination of the interface for collecting EEG signals, EEG components, and signal postprocessing. Furthermore, the paper describes the development of natural interaction strategies, with a focus on signal acquisition, data processing, pattern recognition algorithms, and control techniques.

## 1. Introduction

Stroke has become one of the main reasons for abnormal human death. According to global disease research records, more than 10 million patients worldwide suffer from stroke and up to 116 million people are left with disabilities. This disability affects the patient and the patient’s family’s daily life [1]. Stroke causes damage to the central nervous system. One of the highly predicted injuries is the loss of limb motion. Rehabilitation training is critical for stroke patients. In recent years, there has been a noticeable increase in the survival rate of stroke cases. However, there is still a high demand for advanced rehabilitation methods to speed up the recovery period and improve motor recovery in post-stroke patients, which gives more availability to the concept of brain computer interface BCI rehabilitation systems. Brain-computer interfaces have been given priority usage over the conventional neuromuscular pathways because they enable stroke patients to communicate with the surrounding environment using their brain signals, overcoming the movement disability of the limbs [2]. This advantage caused a growing attraction in the field of rehabilitation. Additionally, the ability to decode the desires of patients diagnosed with motor disability has governed the usage of an external rehabilitative or assistive device. It has proved the ability of BCI systems to apply the neural plasticity concept using neurofeedback [3,4,5].

Furthermore, the BCI rehabilitation system has a great advantage over traditional rehabilitation (e.g., physiotherapist restricted induced therapy movement); the BCI rehabilitation systems are closed-loop, patient-oriented, and stimulate motion. There is no need for the remaining paralyzed limbs to move [6]. Many clinical studies stated a remarkable enhancement in motor recovery by using BCI rehabilitation systems. Furthermore, recent review articles stated that for stroke patients’ rehabilitation methods, clinical research using the BCI rehabilitation system recorded higher clinical scores under controlled conditions [7,8,9]. Although all of these are inspiring advantages for the BCI rehabilitation systems, there are some remaining obstacles, such as the accuracy of the patient detected, motor intention, system stability cross subjects, and the different rehabilitation sessions in the accuracy of real time and real-time brain data processing techniques [10,11,12,13]. Therefore, there is an urgent need to devolve innovative BCI paradigms to further improve the practicability and effectiveness of BCI rehabilitation systems.

There are two main techniques, invasive and non-invasive BCI systems, that measure the subject’s intention by collecting the brain signals. Electrocorticography (ECoG) and electroencephalography (EEG) have risen to prominence as the most often used invasive and non-invasive methods, respectively [14,15]. Electrocorticography (ECoG) of the brain employs single neuron action potentials (single units), multiunit activity (MUA), and local field potentials (LFP) [16,17]. These approaches successfully use these signals’ high-quality spatial and temporal properties to decode biomechanical parameters [18]. However, invasive electrodes have substantial disadvantages due to the danger of undergoing surgery and the progressive deterioration of recorded data [19]. Therefore, non-invasive techniques became more common in human subjects. The non-invasive techniques are magnetoencephalography (MEG), near-infrared spectroscopy (NIRS), functional magnetic resonance imaging (fMRI), (MRI) and electroencephalography (EEG).

Whatever the method used, invasive or non-invasive, after the rehabilitation process it is very important to monitor the effects of rehabilitation. Diffusion tensor imaging (DTI) is a powerful method that allows the investigation of the microstructure of tissues both in vivo and noninvasively [20]. Also, it is a complementary way that can help verify the quality and effects of rehabilitation treatment with the use of a technique that eliminates the impact of systematic errors (BSD-DTI) in the context of supporting the verification of the rehabilitation process. A good DTI can show whether neural fiber tracts are being restored or not.

**Figure 1:**
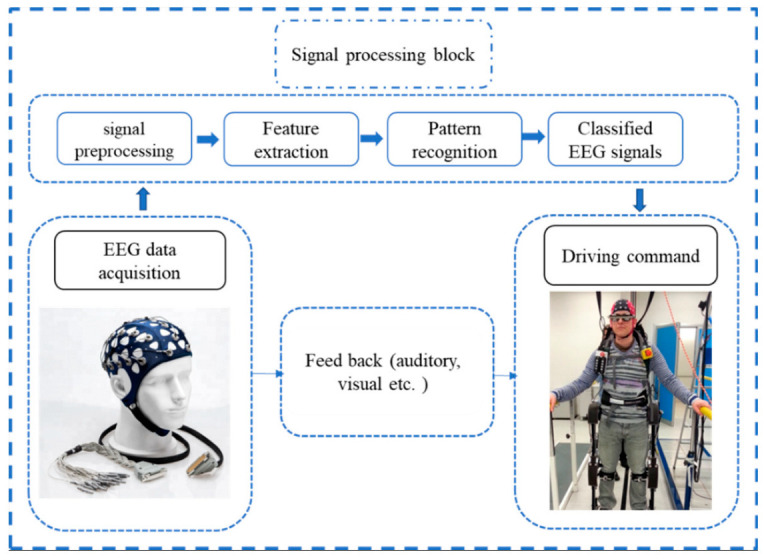
The rule of the EEG signal as the main element in the BCI-EEG rehabilitation system.

Furthermore, new technology advances such as wireless recording, real-time temporal resolution, and machine learning analysis have sparked a surge in interest in non-invasive technologies, particularly EEG-based BCI approaches. Furthermore, EEG is considered the most non-invasive, realistic, and practical brain machine interface technique because other techniques are not portable, expensive and technically challenging. EEG has proven to be the most preferred approach because of its direct measurements of cerebral activity, low cost, mobility for clinical usage, ease to use, and adaptability to multiple experiment paradigms [14]. EEG signals could act as a connection between the brain and various external devices, leading to brain-controlled assistive and rehabilitation devices for disabled people and patients with strokes and other neurological deficits [21]. The BCI-EEG based solution mainly depends on the EEG signal properties and the EEG signal and the processing of the EEG signal, as shown in Figure 1, since the strength of the signal and its subsequent processing affect the accuracy of the controlled rehabilitation device.

Illiteracy in EEG-BCI may impede the wide spread of the EEG-BCI application. An illiteracy of EEG-BCI means that (about 20% of patients) cannot use the EEG signals to control a BCI system, which may impede the broad application of the EEG-BCI technology [22]. In 2015 K. Ang et al. [23] reported that 103 of 125 stroke patients successfully used EEG to modulate and control the BCI neurorehabilitation system, which could prove the practicability of EEG-BCI rehabilitation systems for stroke patients during the rehabilitation sessions [23]. 

A suitable paradigm and protocol should be carefully established for all experiment phases to construct an EEG-based BCI system for a given application. Each of these paradigms has advantages and drawbacks with respect to the patient’s physical state and user friendliness. In each paradigm, the participant performs a specific endogenous and exogenous job (e.g., imaging or visual task) to learn how to modify their brain activity during capturing the EEG signals from the scalp. A neural decoder for the paradigm is created using recorded EEG activity as training data. Following that, the individual repeats the task; then the neural decoder is employed for BCI control. 

BCI systems have been used in two directions. The first is looking at brain activity to see whether a feed-forward route can be utilized to control external devices without targeting rehabilitation [12]. During neurorehabilitation, the other dominating path is closed-loop BCI systems, with the feedback loop playing a critical role in restoring neural plasticity training or regulating brain processes [12]. A suitable paradigm and protocol should be chosen for all experiment parts to develop an EEG-based BCI system for a given application. First, the participant completes a task (e.g., imaging or a visual task) to learn how to modify brain activity while transmitting EEG data. A neural decoder for the paradigm is created using the obtained EEG signals as training data. After that, the individual repeats the task while the neural decoder is used to operate the BCI.

**Figure 2:**
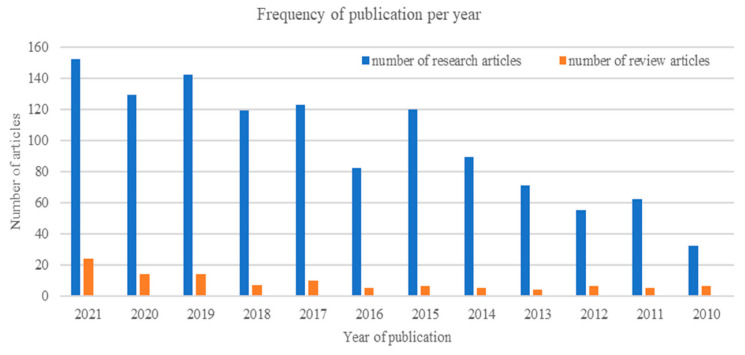
The annual rating of research and review articles on BCI rehabilitation devices.

Limb dysfunction is one of the most common symptoms of stroke patients; according to recent statistics, 80% of stroke patients suffer from limb dysfunction [24,25], affecting the patient and the patient family’s daily life. Moreover, it greatly impacts the patient’s self-trust and economic and social life, and the country’s labor power. For all the factors mentioned above, finding an innovative rehabilitation method becomes an urgent need to replace the traditional rehabilitation method given its weakness, which mainly is slow restoring of the motor function, and finding a rehabilitation method with no need of the dysfunctional limb contribution. Due to the plasticity of the central nervous system, with repetitive rehabilitation training based on brain signal, the brain can establish a new connection with the dysfunctional limb and the central nervous system, which can positively help treat stroke patients. The role of BCI systems is to help patients do practical rehabilitation training for the dysfunctional limb without the need for any contribution of this limb, which is a crucial point for stroke and post-stroke patients. As a result of the problems of traditional rehabilitation, different rehabilitation robotic systems have been built to help patients complete repetitive training through external rehabilitation robots combined with BCI systems [26,27]. Due to the continuous research in rehabilitation robots, the United States and European medical BCI systems markets are continuously growing. More stroke patients with hand dysfunction benefit from rehabilitation robotics with BCI systems. As shown in Figure 2, the annual trend of research publications related to BCI systems and rehabilitation robots in the web of Science database indicates the promising results of the BCI systems. Moreover, the scarcity of review papers shows a lack of reviews that might help the researcher better understand the field and keep up with the new trends and achievements in the field. 

**Figure 3:**
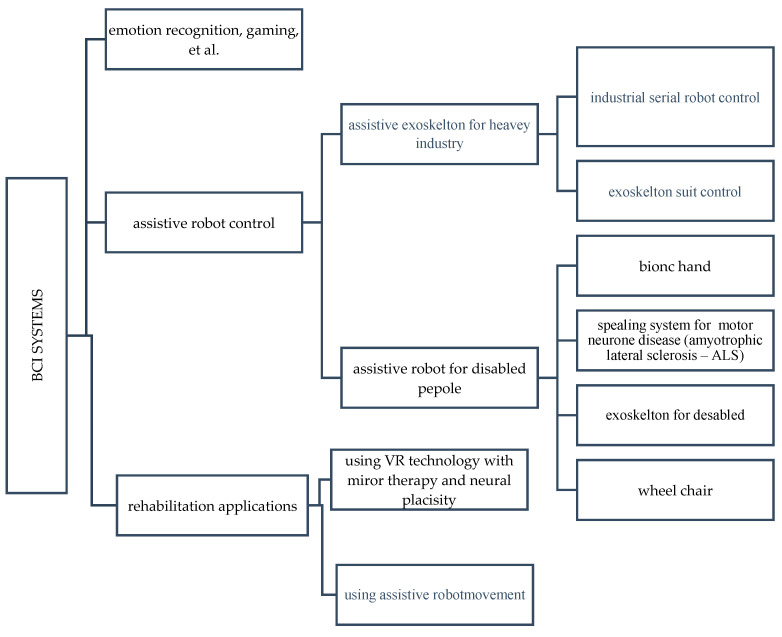
The different division of BCI systems based on the applications and products examples.

Several review papers have been published on the BCI rehabilitation system [28,29,30,31]. But few of these papers have described in detail the applications of the BCI system in different fields, how the BCI concept was applied to these systems, the drive modes, and the control strategies. In this paper, we review the current development of the BCI systems over the last five years and provide an overview of the electroencephalography (EEG), BCI, different paradigms based on exogenous and endogenous EEG signals and their advantages and disadvantages, together with endogenous and exogenous suitability for the different applications besides the control strategies, starting with the EEG signal Pre-processing and classification. Finally, we reviewed the latest BCI application in rehabilitation based on the assistive robot, and the virtual reality technology. Figure 3 explains the various applications of the BCI systems and shows the importance of the BCI in different life sectors, such as gaming, rehabilitation, and in industry. The rest of the paper is organized as follows: Section 2.1 describes the EEG signals and their characteristics. Section 2.2 describes the different paradigms used in the BCI systems based on endogenous and exogenous EEG signal reactions. Section 3.1 and Section 3.2 describe the control strategies, including signal processing and the classification methods for the EEG signals. Section 4.1 and Section 4.2 analyze the different BCI systems’ based on assistive robot technology and virtual reality technology state of the art. Finally, the conclusion of the discussion summarizes the whole paper.

## 2. Overview of EEG and BCI

### 2.1. Electroencephalography (EEG)

**Figure 4:**
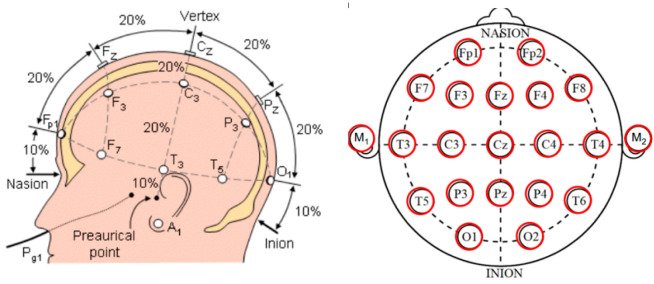
Shows the (10-20) system to place the EEG surface electrode on the scalp [33].

Electroencephalography (EEG) is the most often-used brain signal in brain-machine interface applications. EEG measures brain activity electric signals generated by currents created by neurons within the brain. By placing the electrode on the scalp, the EEG signal can be detected non-invasively [32]; while the electrode placement has different placing systems: (10-5), (10-10), and (10-20) EEG systems; one of the most promising used systems is the (10-20) system. The 10-20 system concept is described as shown in Figure 4. 

Several factors contribute to this popularity compared to other brain wave measurement methods. EEG signals are non-invasive, low cost, compatible, portable, and have a high temporal resolution. This explains why EEG is the most widely used tool to measure brain activity [34]. Furthermore, it is reasonably priced and has an excellent temporal resolution (1 ms). However, it has a poor signal, is prone to artifacts, and has a low spatial signal resolution [35]. Waveforms measured in an EEG test reflect the electrical activity of the brain. The strength of the EEG activity signal is frequently and relatively low, measured in (θ) and gamma [36] depending on their frequency range, as presented in Table 1.

### 2.2. BCI Has Different Paradigms Based on Exogenous and Endogenous EEG Signals 

The BCI system based on EEG signals mainly depends on how the EEG signal is distinguished. It depends on whether the brain ignition method is internal or external. The most used internal and external paradigms are as below: Motor Imagery Paradigms (Imagined Body Kinematics Paradigm, Sensorimotor Rhythms (SMR) Paradigm), External Stimulation Paradigm (Visual P300 Paradigms, Steady-State Visual Evoked Potential Paradigms (SSVEP), Error-Related Potential), Hybrid Paradigms, and others, such as the Discrete Movement Intention Paradigm, the Auditory Paradigm, the Somatosensory (Tactile) paradigm, and the Reflexive Semantic Conditioning Paradigm.

#### 2.2.1. Endogenous EEG Signal

In the endogenous BMI technique, the EEG signal is generated independently of external stimulation and may be fully and freely regulated by the individual. It is also helpful for patients with neurological problems as it allows for more natural and spontaneous interactions because the neuroprosthesis is automatically controlled [51]. However, this often needs longer training time and a lower bit rate than SSVEP and P300. Sensorimotor rhythms (SMR) and slow cortical potentials (SCP) are two examples of endogenous EEG signals [56]. SMR can withstand two types of amplitude modulation: event-related synchronization and event-related desynchronization. Sensorimotor rhythms are composed of m_u_ and beta rhythms, which are different frequencies of brain activity that occur in the mu (7–13 Hz) and beta (13–30 Hz) bands, respectively. In both motor imagery and active motion situations, ERD is indicated by a reduction in EEG power associated with motion-related activities. Sensorimotor rhythms are crucial to motor imagery tasks, even when no effective movement is present [57]. Practicality can be improved using sensorimotor rhythms to create endogenous BCI, which is more helpful than exogenous BCI in practical applications. In their survey, Maged S. AL-Quraish et al. concluded that 57 percent of their selected research employed motor imagery tasks. ERD is the most prevalent signal that has been used as an example in the following studies [58,59,60,61,62,63] to operate assistive devices. Movement-Related Cortical Potentials (MRCPs) indicate fundamental processes proportional to motor execution and are connected to both active and imagined motor tasks [64]. Xu et al. [65] MRCPs’ reliance on force-related characteristics that can be used to generate control signals for impaired people controlling assistive robots also used MRCPs to identify imaginary ankle dorsiflexion motions with a short delay from the scalp EEG. MRCPs may be linked to EEG modules to regulate the ambulation of the lower extremity exoskeleton while walking, as demonstrated in [66].

#### 2.2.2. Exogenous Evoked Potentials, EEG Signal

Exogenous BMI and EEG signals are produced in response to external inputs such as visual or auditory cues. External stimuli, such as flashing LEDs and music, can influence brain activity. The changed EEG activity was recorded and processed to control the actual or virtual items. External stimulation can take the form of visual [67,68], auditory [68,69], or somatosensory [70] stimulation. Visual P300 Paradigms and Steady State Visual Evoked Potential are the most popular forms of External Stimulation Paradigms.

This technique has the advantage of requiring little subject training and achieving a high bit rate of 60 bits/min [71]. However, users must always focus on the external cue or stimulus, which limits its application. Furthermore, due to the overwhelming stimulation, users might soon feel fatigued [72]. Exogenous EEG signals include SSVEP (steady-state visual-evoked potential) and P300 [73]. SSVEP is the natural reaction to visual stimuli at different frequencies [74]. In other words, when a person looks at a flashing light at a given frequency, the visual cortex responds with an EEG signal of the same frequency. SSVEPs are used in exoskeleton robots to provide control signals to the exoskeleton. On the screen, the user is presented with several control inputs, such as moving forward and moving left, which the subject may pick by focusing on. For example, Kwak et al. [75] used a visual stimulation screen with five LEDs attached to the exoskeleton to stimulate SSVEP. Each LED denotes a different control instruction (for example, standing, walking ahead, and turning left/right). Another type of exogenous EEG signal is the P300, which occurs approximately 300 milliseconds after the subject notices an external stimulus. P300, like the SSVEP, is programmed to select one of several potential instructions from which the user intends to trigger a P300 reaction. The P300 does not need any training; however, it has a slower data transfer rate than SSVEP.

## 3. EEG Control Strategies

Different control mechanisms for controlling human-robot interaction have been established. The first approach is to employ a control scheme that predicts or follows the subject’s intention based on data acquired from the exoskeletons. Only the information acquired from the exoskeleton would be used to predict the user’s movement intention in this control method. Two closed loops are needed to control this scheme. The first controller reflects the user and actuator’s effect on the exoskeleton [51]. The second control strategy utilizes a control scheme based on the interaction force that can be measured by measuring the deformation of an elastic transmission element or structure coupled to an exoskeleton robot link. Low-level control techniques (direct control) were used to a large extent in this previously demonstrated electroencephalography (EEG)-based computer interface (BCI)-controlled robotic arm system. However, users must issue control orders often under a low-level control method, which can lead to the user [76]. EEG is a popular non-invasive technology for capturing brain activity. EEG signals are analyzed and translated into control commands [77]. The interface between the human and the wearable robot is crucial for an efficient and successful control scheme that predicts the intention of the user to move. Consequently, the control scheme can be categorized according to the human-robot interaction. Exoskeleton information is obtained based on the interaction force measured between the exoskeleton and the human. The physiological signal measured from the human body reflects the user’s movement intention, Electroencephalography (EEG) signals significantly impact the development of assistive rehabilitation devices. EEG signals have recently been employed as a common way to explore human motion behavior and functions [51]. Human motion intention (HMI) based on EEG can control different kinds of robots to assist paralyzed persons with neuromuscular diseases such as amyotrophic lateral sclerosis and stroke in rehabilitation training. Compared to the traditional approach of repeated motion, a large body of research suggests that EEG-based assisted robots enhance patients’ recovery by essentially helping to reestablish the neural circuit between the brain and the muscles [78]. Brain potentials captured by scalp electrodes are converted into commands for controlling robot arms, exoskeletons, wheelchairs, or other robots through brain-computer interface algorithms. Slow cortical potentials, event-related P300, and steady-state visual evoked potentials are several EEG processes that distinguish EEG-based brain-computer interfaces [77]. In terms of reliability, the BCI can be divided into independent BCI and independent BCI. The dependent brain-computer interface enables people to use some form of motion control, such as gaze. The brain-computer interface based on moving images is one of the most commonly-used paradigms of brain-computer interface [79]. Independent BCIs such as P300 evoked potentials, steady-state visual evoked potentials (SSVEPs), sensorimotor rhythms, motion-onset visual evoked potentials, and slow cortical potentials can be utilized to extract control signals; SSVEPs are periodic evoked potentials (PEPs) generated by rapidly repeating visual stimulation, particularly at a frequency greater than 6 Hz. The 5–20 Hz stimulation frequencies produce the most significant response to visual inputs. SSVEPs are more abundant in the occipital and parietal lobes, and their frequency corresponds to the fundamental frequency and harmonics of the frequency-coded inputs. By extracting frequency information from this signal, an SSVEP-BCI system may identify the user’s intended command, such as moving a cursor on a computer screen or operating a robot arm [80]. SSVEP-based BCIs have a high information transfer rate (ITR) and need little user training. SSVEP-based BCIs are easier to encode with more instructions without much training and show good promise in high-speed communication [76] but are limited by a small number of controls. In other words, SSVEP-BCIs of various classes can be realized using flickering lights with different frequencies. These flashing stimuli, given using light-emitting diodes (LEDs) or a computer display, change EEG signals at the stimulating frequency and its harmonics. The frequency components of SSVEP could be calculated using the lock-in analyzer system (LAS), the power spectral density analysis, and the canonical correlation analysis (CCA) [81].

BCIs based on SSVEP and the P300 component can be set up with little or no training, but they require external stimuli. In contrast, BCIs based on sensorimotor rhythms (SMR) and slow cortical potentials (SCP), on the other hand, do not require external input but do require significant user training [81]. The assist robot can be controlled more easily via event-related potentials (ERPs), which are brain voltage fluctuations reacting to certain stimuli such as sights or noise. A lower limb prosthesis based on P300, the peak detected 300 ms (250–500 ms) following a given event, has been developed to assist persons in walking. Motor imaging (MI) has also been addressed to tightly connecting brain commands and bodily movement responses. A method for after-stroke rehabilitation activities that use MI to control a robot to drive the arm by allowing individuals to visualize moving their hands has been demonstrated [77]. Because of its effectiveness over traditional BCI, hybrid BCIs (hBCI), which “detect at least two brain modalities in a simultaneous or sequential pattern,” have been emphasized for control applications [82]. Researchers looked for multiple regions of the brain to boost the number of commands, improve classification accuracy, reduce signal detection time, and shorten brain command detection time. For example, SSVEPs and event-related potentials (ERPs) were mixed to generate a hybrid EEG paradigm. The combination of SSVEP and P300 signals for BCI is a good example. SSVEP has also been paired with motor imagery (MI) [83]. EEG is also hybridized with electrooculography (EOG), functional near infrared spectroscopy (fNIRS), electromyography (EMG), and eye tracker [84].

### 3.1. EEG Signal Preparation Overview

**Figure 5:**
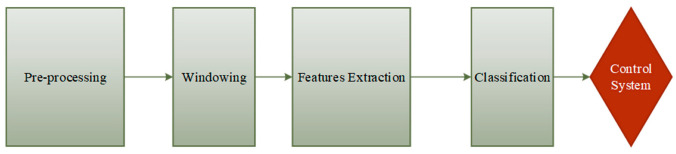
EEG signal processing for HMI.

To operate external devices such as an upper or lower limb exoskeleton using an EEG signal, the individual must generate various brain activity patterns (motor imagery or motor execution), which will be identified and translated into control commands [84]. The detected brain signal is preprocessed to remove artifacts and prepare the signal for machine learning, turning EEG signals into control commands operating terminal devices. The feature extraction stage began from this process, and the extracted features were then submitted to a feature reduction procedure if necessary. Finally, the new projected feature vectors are divided into various classes based on the task. A BCI system has four major components: signal acquisition, signal preprocessing, feature extraction, and classification, as depicted in Figure 5.

The user conducts MI of the limb, which is encoded in EEG readings; features describing the task are deciphered, processed, and transformed into commands to control the assistive robot equipment.

The brain signals are captured in the signal acquisition stage, which may also include noise reduction and artifact processing. Skin impedance fluctuations, electrooculography activity, eye blinks, electrocardiographic activity, facial/body muscular EMG activity, and respiration can cause EEG abnormalities. The bandpass filter can be an effective preprocessing tool because the frequency ranges for the physiological signals are typically known.

### 3.2. Feature Extraction

The feature extraction stage is to identify distinguishable information in the recorded brain signals. Then, the EEG signals can be mapped to different processing vectors, which include the actual features and discrimination features of the measured observation signals. Some methods divide the signals into short parts, from which the parameters can be calculated. The length of the segment length impacts the accuracy of the estimated features. Wavelet transform or adaptive autoregressive components are preferred to highlight non-stationary time changes in brain signals [14].

Several distinct feature extraction techniques, including the autoregressive model, discrete wavelet transform, wavelet packet transform, and sample entropy, were utilized. The redundant and irrelevant information was managed by the feature selection methods, which benefited classification. To improve the performance of feature selection, one of the global optimization strategies based on binary particle swarm optimization (BPSO) is presented [85,86]. To evaluate the efficacy of feature extraction, class separability experiments are conducted. Using a 14-channel EEG machine, 21 healthy subjects aged 12 to 14 years who viewed images containing one of four distinct emotional stimuli had scalp EEG data recorded (happy, calm, sad or scared). 

Then, a balanced one-way ANOVA was used to determine the most useful EEG characteristics. Statistics-based selection of features outperformed manual or multiple variable selection. Support vector machine, k-nearest neighbor, linear discriminant analysis, naive Bayes, random forest, deep learning, and four ensembles were used to classify emotions using the most effective features [87,88]. In addition, a Markov is employed to process the simulated EEG signals based on the actual EEG signals. Simulated and experimental results demonstrate that the performance of the proposed method is superior to that of widely used methods [89,90]. The proposed method can prevent the mixing of components of EEG signals with complex structures and extract brain rhythm from EEG signals with low SNR.

The most common features of EEG-based BCIs include spatial filtering, band power, time points, etc. [91]. In addition, stationary subspace analysis (SSA), which decomposes multivariate time series into stationary and non-stationary components, has recently been presented to cope with the non-stationarity of EEG data [14] after the retrieved feature vector is used to train a classifier [92].

### 3.3. Classification 

Small changes can easily affect the complex structure of EEG in human cognition. As a result, a highly efficient and robust classifier is required. In a BCI system, the objective of the classification step is to recognize a user’s intents using a feature vector that characterizes the brain activity provided by the feature step. This goal can be achieved using regression or classification methods. However, classification techniques are currently the most preferred option. Regression methods use features retrieved from EEG signals as independent variables to predict user intentions. On the other hand, classification algorithms use the extracted features as independent variables to create boundaries between various targets in the feature space [14].

Classification algorithms turn the extracted data into distinct motor activities such as hand gestures, foot movements, word production, and so on in motor imagery brain-computer interfaces [79]. Combining several signal characteristics from different modalities/devices for the same brain activity can increase the classification accuracy. For example, finger-tapping and hand/arm movement have been detected using a combination of EEG and fNIRS [84]. Machine learning (ML) and deep learning (DL) techniques have been used to identify EEG-based BCI; with each successive session, machine learning techniques allow the brain-computer interface to learn from the subject’s brain, modifying the generated rules for classifying ideas and thereby increasing the effectiveness of the system [79]. Machine learning algorithms are divided into three groups based on their results: supervised, unsupervised, and reinforcement learning [79]. Moreover, deep learning approaches have been shown to improve classification accuracy. Deep networks can also detect latent structures or patterns in raw data [92], and robots can study innate movement patterns and estimate human intentions when combined with MLAs [93].

Various classification algorithms have been implemented, such as k-nearest neighbors (k-NN), multilayer perceptron (MLP), decision trees [92], convolutional neural network (CNN) [83], linear discriminant analysis (LDA), support vector machine (SVM) [79] with the SVM classifier outperforming other classifiers such as LDA and K-NN [79]. When comparing the classification accuracies of LDA, SVM, and backpropagation neural network (BPNN), the former two classifiers produced similar high accuracies, which are more significant than BPNN [59]. Compared with PCA, Recurrent Neural Network (RNN) obtained a control accuracy of 94.5 percent and a time cost of 0.61, whereas the PCA algorithm achieved a control accuracy of 93.1 percent and a time cost of 0.48 ms [94].

A convolutional neural network (CNN) based deep learning framework is employed for inter-subject continuous decoding of MI-related electroencephalographic (EEG) signals. The results, which were obtained using the publicly available BCI competition IV-2b dataset, show that adaptive moment estimation and stochastic gradient descent yield an average continuous decoding accuracy of 71.49 percent (a = 0.42) and 70.84 percent (=0.42) for the two different training methods, respectively [95,96].

The pattern recognition step is coming after the feature classification step, which means that the EEG signal has been classified into different shapes, and the subsequent step is required to determine the pattern recognition. This is the case for this part of the process. Statistical data analysis, signal processing, image analysis, information retrieval, bioinformatics, data compression, computer graphics, and machine learning are just some of the fields that can benefit from its use. The fields of statistics and engineering are where pattern recognition first found its roots; some contemporary approaches to pattern recognition include the application of machine learning.

## 4. Application of EEG in BCI Systems 

**Figure 6:**
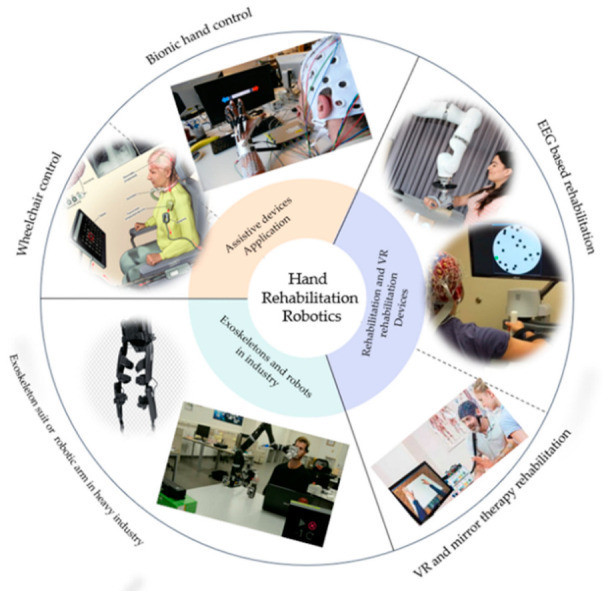
Application of the BCI system.

EEG signals can offer a channel from the brain to several external devices, providing a brain-controlled assistive device for disabled and brain-controlled rehabilitation equipment for patients with stroke and neurological abnormalities [97,98]. Control equipment such as wheelchairs [97] and communication help systems [98] have been programmed using EEG signals, as shown in Figure 6. Throughout the last decade, different EEG approaches have proven a viable strategy in controlling rehabilitative and assistive equipment.

### 4.1. BCI-Assistive Robot Rehabilitation Application

**Figure 7:**
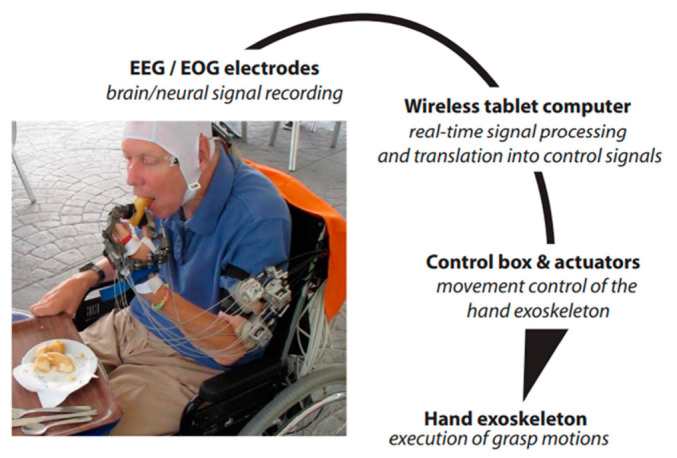
Scheme of the process pipeline to control the exoskeleton of the hand. Adapted with permission from Ref. [99]. 2022, S.R. Soekadar et al.

In 2016, Applied Neurotechnology Laboratory, University Hospital Tübingen, Germany, used a hybrid non-invasive neural hand exoskeleton with six paraplegic subjects aged between 30 ± 14 to control the paralyzed wrist fingers flexion/extension movement, as shown in Figure 7. They controlled the hand exoskeleton by wireless transmitting of the EOG and EEG signals to a tablet that did the signal preprocessing and converted the final signal record into a control command, thus sending it to the control box and then to the actuator to move the hand mechanism using a flexible cable system. Their system proved that the assistive brain/neural systems could help the paraplegic patients independently do their daily activities, such as holding a cup and drinking, eating with cutlery or manipulating different objects [99].

**Figure 8:**
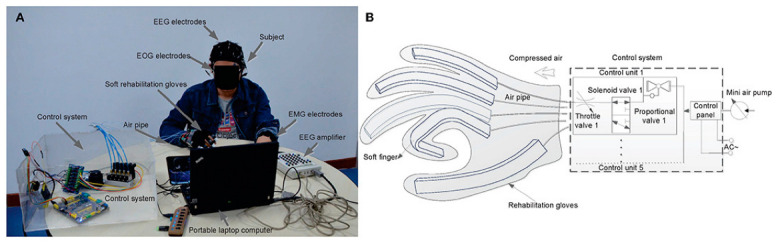
(**A**) shows the prototype model BCI and its experimental condition, (**B**) is the control scheme of the soft robot hand [100].

**Figure 9:**
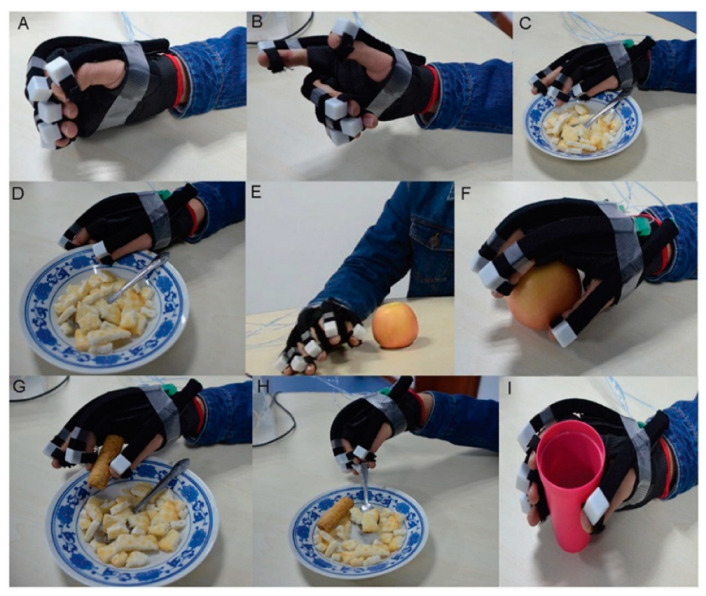
(**A**–**I**) are the results presentation of the hand action. As an example, A is grasping various objects in their everyday life quickly according to his/her intention with the help of a soft robot [100].

In 2019, Zhang Jinhua et al. considering the needs of hand rehabilitation, as shown in Figure 8, they created a multimodal human-machine interface system using three bio-signals which are electroencephalography (EEG), electrooculography (EOG), and electromyogram (EMG). They use bio-signals to generate a multi-control command for a multitask, real-time soft assistive robot; moreover, they investigated the acceptance of the patient of use of a wearable hand for a robot assistive hand movement, as shown in Figure 9. To apply the concept of using EEG, EOG, and EMG together, six subjects were hired to experiment with imaginary flow motor for EEG, looking left/right for EOG, and different hand movements for EMG. The subject spent <2 min training to set the EEG/EOG mode parameters. The experiment scenario was as follows: 2 s black screen, then there is a cross on the screen center and lasts until 4 s; after that, a cue picture in a dashed border appeared for 2 s. For the EOG, a left or right arrow appears on the screen to guide the subject to track the arrow by looking left/right, which then makes the subject blink. The EEG mode was imagining the left/right-hand movement on the screen as a cue for 2 s. For training, the EOG and EEG models contained ten trials, including five left and five right arrows, five times left hand, and five times right hand MI. Using this model, the number of control commands that could be achieved is significantly greater than in other single modes. This multifunction had achieved a classification precision of 93.83% with a 47.41 bit/min information transfer rate, which means the user can control 17 action/minute, which is convenient for disabled patients [100].

**Figure 10:**
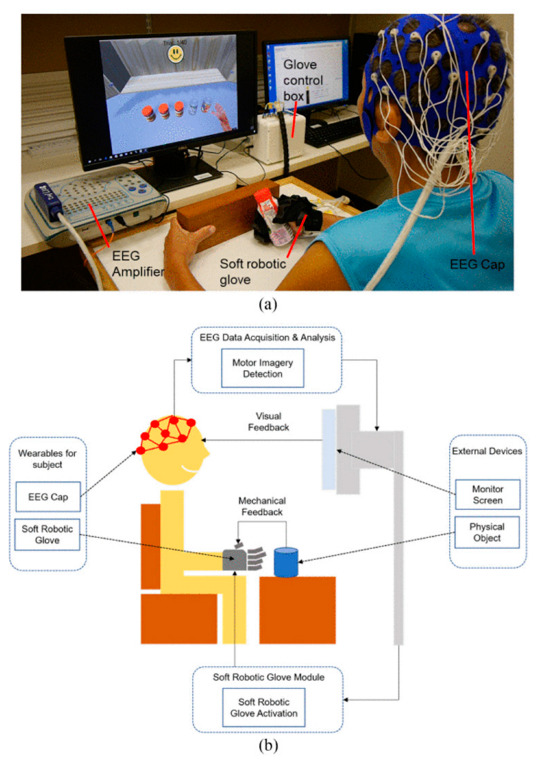
The setup of BCI-assisted soft robotic glove for stroke rehabilitation at (**a**) a local hospital, with (**b**) depicting an illustrated overview. The setup comprises a EEG cap, EEG amplifier, and soft robotic glove. Adapted with permission from Ref. [101]. 2022, Cheng N et al.

In 2020, N. Cheng et al. studied the effect of BCI-based Soft Robotic Gloves on the rehabilitation progress compared to the soft robotics glove for stroke patient rehabilitation. A total of 11 chronic stroke patients were recruited for the 24 weeks experiment and divided with two groups (six patients in the BCI soft robotic glove) and five patients in the Soft robotic glove to do daily life-oriented tasks. The BCI-with soft robotics glove group used BCI motor imagery-based. The soft robotics glove group used the soft robotic glove to assist the patient affected hand in daily activity tasks. In the BCI- soft robotic glove. The patient is provided with the intended task through a computer screen, performing motor imagery by imagining the task. Then the subject’s EEG signal is collected using the EEG cap, and ERD/ERS is detected from the patient’s EEG signals. The acquisition system sends two control signals, one to the robotic glove to activate the actuator and assist the hand in performing finger-specific movement tasks, and the other to the computer screen to play an animation for a successful specific hand movement task, as shown in Figure 10. During the first six weeks, the two groups had no significant changes. But after that, all patients with the BCI soft robotic glove announced a sense of small movement of the stroke-impaired hand, while this sense lasted with three out of five of these patients until the 24th week. None of the patients with only the soft robotic glove had this sense during the 24 weeks. Their results indicated that BCI combined with soft robotic training for ADL-oriented stroke recovery has the potential to provide long-term benefits and prompt perception of motor actions [101].

**Figure 11:**
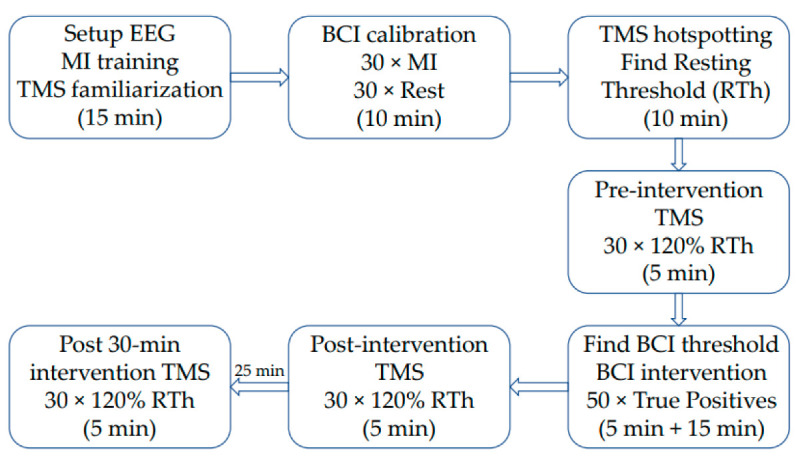
Timeline of the experiment [102].

In 2021, Mads Jochumsen et al. developed a cheap BCI system with a cheap 3D-printed wrist exoskeleton controlled with an open source cheap BCI. The aim was to overcome the major obstacle that impedes BCI use in the rehabilitation field, which is cost and usability, and to check if its system can simulate neural plasticity. Eleven healthy subjects joined this experiment, including four females aged 28 ± 3. EEG was recorded from seven channels (OpenBCI, Brooklyn, NY, USA) from F1, F2, C3, Cz, C4, P1, and P2 concerning the International 10-20 System. The subjects were seated in a chair and it was explained how to do motor imagination and then there was a training session for 5 min. Subjects were asked to follow the procedure to image 30 extensions of the right-hand wrist extensions with a specific timeline; see Figure 10 for the experiment timeline, as shown in Figure 11 [102]. 

During the experiment, the subject was instructed through a screen and the visual cue was as follows, every 30 successful motors imaginary (display “REST”) and 30 motor imagines were recorded. The imaginary right hand wrist movement lasts for 4 s. The subject wore the wrist exoskeleton on the right forearm and hand during the experiment. The subject’s forearm was comfortably on the chair armrest. The experiment was completed successfully if a 50 right exoskeleton movement was obtained due to 50 right-hand wrist imagination. The TMS was measured before, immediately after, and 30 min after BCI training. They found that the BCI system has an 86.12% true positive rate and 1.20 0.57 false detections per minute. The MEPs increased by 35–60% immediately after the BCI training and 67–60% 30 min later than the measurement before the BCI training. There was no correlation between BCI performance and plasticity induction.

**Figure 12:**
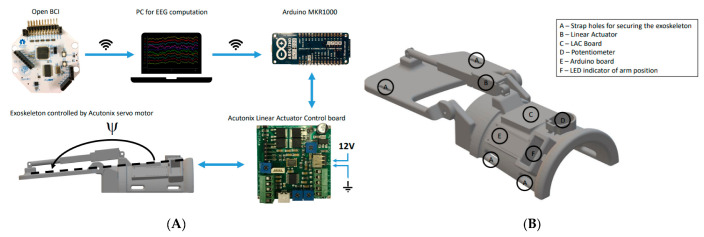
(**A**) The hardware setup. The Arduino control board and Linear Actuator were mounted on the exoskeleton. The signals were transferred to the OpenViBE PC running OpenViBE via a wireless connection. When an imagined wrist extension was identified, a trigger was transmitted to the Arduino on the exoskeleton through wireless communication. A wire linked the Arduino to the Linear Actuator Control board. A 12 V power supply was used to power the Linear Actuator Control board. A wire linked the motor to the Linear Actuator Control board. (**B**) The 3D-printed exoskeleton. The contact surfaces of the forearm were padded with foam to improve comfort. Velcro straps were used to fix the exoskeleton to the subject’s forearm [102].

In summary, an open-source BCI system can detect imagined motions and operate a low-cost 3D printed exoskeleton, which may induce brain plasticity when paired with the BCI. Their discoveries might help BCI technology to become widely used in rehabilitation at home, as shown in Figure 12. However, users must be enhanced, and further experiments with stroke subjects are required [102].

### 4.2. BCI-Virtual Reality Rehabilitation Application

**Figure 13:**
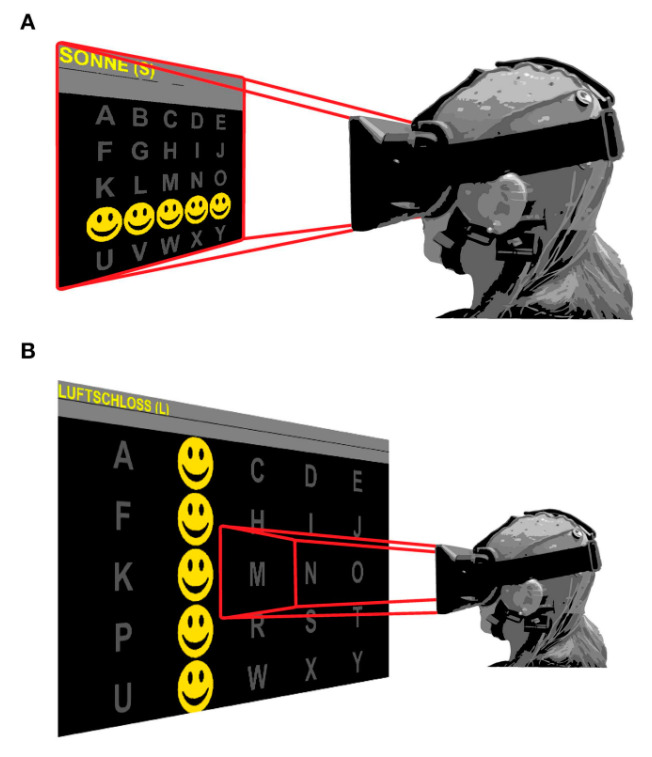
(**A**) Field of view for the glasses. In (**A**), the user sees the five-by-five matrix and the screen is fixed. In (**B**), the user can see only one letter from the five-by-five matrix, and the subject moves his head to concentrate on a specific letter [103].

In 2015, Kathner et al. researched whether VR devices can achieve the same precision and rapid data transmission compared to regular display methods that are used in the P300-BCI systems. They conducted an experiment based on 18 subjects who were asked to do an online spelling task using three different presentation methods, as shown in Figure 13. The first screen was a standard thin film transistor five-by-five matrix. The second was the same five-by-five screen but in a virtual reality scenario that filled the subject’s field of view. The third was similar in VR, but only one letter from the five-by-=five matrix filled the subject’s field of view at a time. Empirical findings revealed equivalent online spelling accuracy (96%, 96%, and 94%, respectively). As a result, VR devices could report the same precision as regular flat panel displays while still performing quick P300-BCI transmission [103].

Ortner et al. from Johannes Kepler University Linz, Austria found that fusion of VR systems and MI-based BCI can improve the efficiency of rehabilitation training for patients with neurological diseases, particularly motor impairment. The proposed MI can be a common technique for motor rehabilitation of stroke patients by using the MI-BCI system to train stroke patients to imagine left- and right-hand movements in VR scenes. The researchers also optimized an algorithm that decreased the classification average error to 9.6% [104]. Moreover, other researchers provided additional evidence that neurological disease patients can use motor imaginary based BCI systems to imagine and run a virtual or actual device in VR scenes to perform and repeat different movements as rehabilitation training sessions, therefore performing a training aim to neural plasticity and helping the recovery of the injured motor nerve pathway.

**Figure 14:**
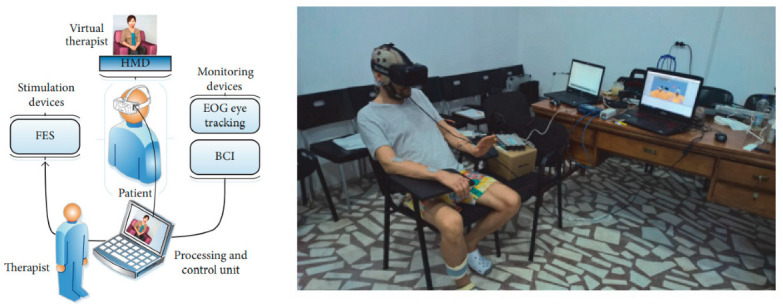
BCI-FES subsystem and the patient executing the control command for a rehabilitation exercise [105].

In 2018, Robert Lupu et al., Technical University of Iasi, Romania presented a stroke rehabilitation therapy method based on a novel technique. The suggested approach places the patient in a virtual situation where a virtual therapist organizes activities to restore brain function using a virtual reality Oculus Rif device. The electrical stimulator assists the patient in rehabilitation activities, and a BCI system and an EEG device are used to verify whether the exercises are being performed correctly [105]. The BCI-FES TRAVEE subsystem consists of a stimulation part which is FES, BCI monitoring devices, an electro-oculography (EOG), a VR headset that is Oculus Rif, and a computer, as shown in Figure 14 [105].

**Figure 15:**
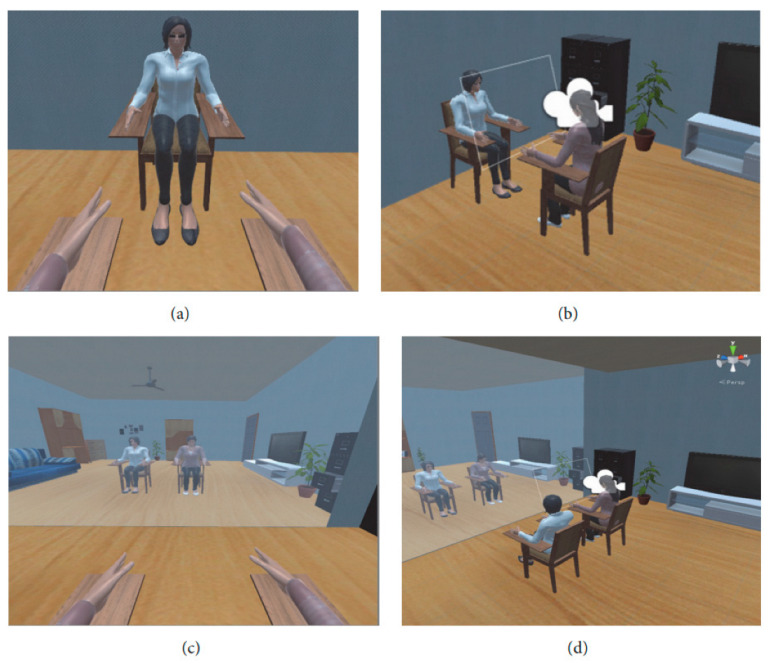
The VR environment: (**a**,**c**) patient views; (**b**,**d**) world views; (**a**) the therapist in front of the patient; (**c**) the therapist on the left side of the patient with mirror in the front [105].

This system was focused on flexion and extension. The experiment scenario was as follows: the patient seated in a normal chair or wheelchair. FES electrodes were placed on the forearm extensor muscles as shown in Figure 13. The EEG helmet and EOG electrodes are placed before attaching the VR headset. The therapist sits in front of the patient and shows him the following images to describe what he will see: The virtual therapist will raise their hand, as shown in Figure 15 (the therapist’s left hand is the patient’s right hand); a large arrow will appear on the upper left or right of the screen, depending on the virtual therapist’s indications, and the patient will also hear sounds from the left or right. The following explanations are given: VR is fitted, the EOG system is calibrated, and recovery exercise may begin, but only after the actual therapist informs the patient that he has the option of selecting between two views: frontal perspective (the virtual therapist is located in front of the patient) or a mirror view (the virtual therapist is on the left side, and a mirror is in front of them, as in a dance studio), as shown in Figure 14. In this system, the subject is not disturbed by surrounding events or the real environment. The subject is only immersed in VR, and the VR therapist shows the subject how to do every exercise, and a big red arrow is shown every time. The eye tracking system detects when the patient loses concentration and sends a warning. The system achieved low control error rates compared to the ones they reported in the investigation. Table 2, Summarizes some of the presented paper details such as subject, signal type and electrode location and other details.

## 5. Conclusions

1. The P300-BCI system is convenient for rehabilitation due to its effective cost, reliable performance, and variety of applications. Furthermore, many research groups integrated the P300 with VR technology for rehabilitation of an immersive experience for neurological diseases. MI offers a solid basis for BCI research and implementation, and the combination of MI-based BCI and VR systems increases the effectiveness of rehabilitation training for people with neurological diseases, particularly motor impairment. In VR feedback, there are obstacles in development and implementation. For example, people may struggle to focus on goals while ignoring the immersive virtual world, which can be distracting. Furthermore, the use of VR equipment is not consistent across the duration of experiments. Both characteristics diminish the efficacy of rehabilitation training. Researchers ran tests on several BCI feedback and VR platforms to discover a reliable approach.
2. The most promising paradigm uses the MI-VR novel multiplatform prototype that improves attention by providing multimodal feedback in VR settings utilizing cutting-edge head-mounted displays. By integrating an immersive VR environment, sensory stimulation, and MI, the NeuRow system is a promising VR BCI system that can offer a holistic approach to MI-driven BCI.

## Figures and Tables

**Table 1:** Shows the EEG rhythms and their frequency.

Rhythms	Rhythm Frequency Band (Hz)	Functions Related
Delta (δ)	0.5–4 HZ	appear in infants and deep sleep [37,38,39,40,41,42].
Theta (θ)	4–8 HZ	It occurs in the parietal and temporal areas in children [43,44,45]
Alpha (α)	8–13 HZ	It can be found in a wake adult. It also appears in the occipital area; however, it can be detected in the scalp frontal, and parietal regions [46,47,48].
Beta (β)	13–30 HZ	Decreasing the Beta rhythm reflects movement, planning a movement, imagining a movement, or preparation of movements. This decrease is most dominant in the contralateral motor cortex. These waves occur during movements and can be detected from the central and frontal scalp lope [49,50,51].
Gamma (G)	>30 HZ	It is the higher rhythms that have frequencies of more than 30 Hz. It is related to the formation of ideas, language processing, and various types of learning [52,53,54,55]

**Table 2:** Summarizes some of the presented paper details such as subject signal type and electrode location.

Type of Application	Representative Works	BCI Paradigm	Description	No. of Subjects	Signal Type	Electrode Number	Accuracy
BCI-Assistive robot for Rehabilitation	Soekadar, S R et al. [99]	MI- EEG HOVs’ EOG	Help paraplegic patients to control the exoskeleton hand for daily life activity	6	EEG-EOG	C3	84.96 ± 7.19%
	Zhang Jinhua and et al. [100]	MI-EEG Left/right looking-EOG		6	EEG-EOG-EMG	40 Ag/AgCl channels placed 10–20 System	93.83%
	N. Cheng et al. [101]	MI	Studied BCI-based Soft Robotic Glove applicability for stroke patient rehabilitation in daily life activities.	11	EEG	24 Ag/AgCl channels placed 10–20 System	-
	Mads Jochumsen and et al. [102]	MI	Induction of Neural Plasticity Using a Low-Cost Open Source Brain-Computer Interface and a 3D-Printed Wrist Exoskeleton	11	EEG	F1, F2, C3, Cz, C4, P1, and P2	86 ± 12%;
	Kathner et al. [103]	P300	Check if VR devices can achieve the same precision and rapid data transmission compared to the regular display methods	18 + 1 person (ALS). 80 years	EEG-VR	Fz, Cz, P3, P4, PO7, POz, PO8, Oz	96%
BCI-virtual reality based for rehabilitation	Ortner et al. [104]	MI	training stroke patients to imagine left and right hands movements in VR scenes	3	EEG-VR	63 positions	mean 90.4%
	Robert Lupu et al. [105]	MI	Flow instruction of virtual therapists, to control virtual characters in VR scenes using MI. Motor function was improved.	7	EEG-FES EOG	16 sensorimotor areas of channels sensorimotor areas	mean85.44%

## Data Availability

The data presented in this study are available on request from the corresponding author.
